# Pyrosequencing versus methylation-specific PCR for assessment of MGMT methylation in tumor and blood samples of glioblastoma patients

**DOI:** 10.1038/s41598-019-47642-2

**Published:** 2019-07-31

**Authors:** Anna Estival, Carolina Sanz, Jose-Luis Ramirez, Jose Maria Velarde, Marta Domenech, Cristina Carrato, Ramón de las Peñas, Miguel Gil-Gil, Juan Sepúlveda, Roser Armengol, Isaac Cardiel, Alfonso Berrocal, Raquel Luque, Ana Herrero, Carmen Balana

**Affiliations:** 1 Medical Oncology Service, Catalan Institute of Oncology Badalona, Hospital Germans Trias i Pujol, Applied Research Group in Oncology (B-ARGO Group), Badalona, Spain; 2 Pathology Service, University Hospital Germans Trias i Pujol, Badalona, Spain; 3 Statistical Unit, Applied Research Group in Oncology (B-ARGO Group), Institut Investigació Germans Trias i Pujol (IGTP), Badalona, Spain; 4 Medical Oncology Service, Hospital Provincial de Castellón, Castellón, Spain; 5 Medical Oncology Service, Catalan Institute of Oncology l’Hospitalet de LLobregat, Bellvitge Biomedical Research Institute (IDIBELL) l’Hospitalet de Llobregat, Barcelona, Spain; 6 Medical Oncology Service, University Hospital 12 de Octubre, Madrid, Spain; 7 Neurosurgery Service, Hospital Germans Trias i Pujol, Badalona, Spain; 8 Medical Oncology Service, University Hospital General de Valencia, Valencia, Spain; 9 Medical Oncology Service, University Hospital Virgen de las Nieves, Granada, Spain; 10 Medical Oncology Service, Hospital Miguel Servet, Zaragoza, Spain

**Keywords:** CNS cancer, Predictive markers

## Abstract

Circulating biomarkers in blood may provide an interesting alternative to risky tissue biopsies in the diagnosis and follow-up of glioblastoma patients. We have assessed *MGMT* methylation status in blood and tissue samples from unresected glioblastoma patients who had been included in the randomized GENOM-009 trial. Paired blood and tissue samples were assessed by methylation-specific PCR (MSP) and pyrosequencing (PYR). After establishing the minimum PYR cut-off that could yield a significant difference in overall survival, we assessed the sensitivity, specificity, positive predictive value and negative predictive value (NPV) of the analyses. Methylation could be detected in cfDNA by both MSP and PYR but with low concordance with results in tissue. Sensitivity was low for both methods (31% and 38%, respectively), while specificity was higher for MSP in blood than for PYR in plasma (96% vs 76%) and NPV was similar (56 vs 57%). Concordance of results in tissue by MSP and PYR was 84.3% (P < 0.001) and correlated with outcome. We conclude that detection of cfDNA in the blood of glioblastoma patients can be an alternative when tumor tissue is not available but methods for the detection of cfDNA in blood must improve before it can replace analysis in tumor tissue.

## Introduction

Glioblastoma accounts for the majority of gliomas (56.6%), with an incidence rate of 3.21 cases per 100,000. It is the malignant glial tumor with the worst outcome^1^. Clinical prognostic factors are age, functional and cognitive status, and extent of surgery, where patients with only biopsy have the worst prognosis^2,3^. The standard treatment, established in 2005 and not modified since, consists of maximal surgical excision followed by radiation therapy with concomitant and adjuvant temozolomide (TMZ)^4^. TMZ is a cytotoxic drug that acts as an alkylating agent. Methylation of the promoter of O^6^-methylguanine DNA methyltransferase (*MGMT*) impairs production of the DNA repair enzyme, which enhances the cytotoxic effect of TMZ. *MGMT* methylation is thus an important predictive and prognostic factor of TMZ treatment^5^, and patients with unmethylated (UNMET) *MGMT* seem to gain only a marginal benefit from adding TMZ to radiation therapy. The analysis of *MGMT* methylation is mandatory in clinical trials and is an important element in routine clinical practice when deciding between radiotherapy alone or combined with TMZ in elderly patients^6–8^.

Other molecular alterations also drive the pathogenesis and behavior of glioblastoma and may affect clinical outcome and the sensitivity of tumors to therapy^9–13^. Intense research into these alterations has led to a new World Health Organization (WHO) central nervous system (CNS) tumor classification that incorporates several molecular diagnostic markers in addition to morphological criteria^14^. Furthermore, for a rapid integration of molecular pathogenesis into clinical practice, the Consortium to Inform Molecular and Practical Approaches to CNS Tumor Taxonomy (cIMPACT-NOW), established in 2016, regularly updates information on molecular criteria and clinical outcome^15^.

Over the last few years, there has been a growing interest in the analysis of tumor molecular alterations in body fluids, a practice known as “liquid biopsy”^16–18^. In cell-free DNA (cfDNA), for example, investigators have detected point mutations, microsatellite alterations, chromosomal alterations, and hypermethylation of promoter sequences^19–21^. Liquid biopsies are of special interest in brain tumors for several reasons. Firstly, due to the difficulty in obtaining tissue from tumors located in areas that are eloquent or not easily accessible, such as the brain stem, liquid biopsies are a highly promising diagnostic tool. Secondly, liquid biopsies are a non-invasive method to monitor molecular changes in tumors throughout the evolution of the disease^20,22^. Finally, the problems involved in differentiating real tumor from treatment-related processes, such as radionecrosis, pseudoprogression, pseudoresponse and immune-related events with magnetic resonance imaging (MRI)^23,24^ suggest that liquid biopsies could be useful for monitoring treatment response and detecting recurrence. Molecular alterations in brain tumors have been detected in cerebrospinal fluid (CSF) and in blood (serum/plasma), includingalterations in DNA in exosomes and circulating tumor cells (CTCs), in cfDNA, and in microRNAs^25,26^.

Our group has a long history of analyzing cfDNA in glioblastoma, through the assessment of *MGMT* methylation in blood using methylation-specific PCR (MSP)^27,28^. However, we hypothesized that by using a more standardized and objective method, such as pyrosequencing, we could obtain more accurate results. Therefore, we used MSP and pyrosequencing (PYR) to analyze MGMT methylation both in tumor tissue and in paired blood samples from a homogeneous cohort of unresected glioblastoma patients and compared the results of the four analyses.

## Results

Patients in the present study had been included in the randomized phase II trial GENOM009 (clinicaltrials.gov NCT01102595)^29^. *MGMT* methylation status both in blood and tissue by MSP was a secondary objective in the trial. Of the 102 patients registered in the trial, nine withdrew before starting treatment and had no further follow-up, so they were not included in the outcome analyses. Ninety-three patients were randomized: 45 to receive TMZ and 48 to receive TMZ plus bevacizumab (BEV). Patient characteristics are summarized in Table 1.

**Table 1:** Characteristics of patients registered in the GENOM009 trial (clinicaltrials.gov NCT01102595)^29^.

Characteristic	N = 102 N (%)
**Included in outcome analyses**	
No	9 (8.82%)
Yes	93 (91.2%)
**Treatment arm**	
A: temozolomide	53 (52.0%)
B: temozolomide + bevacizumab	49 (48.0%)
**Age**, **yrs**	
median (range)	63 (36–79)
≥50 years old	97 (95.1%)
<50 years old	5 (4.90%)
**Sex**	
Male	60 (58.8%)
Female	42 (41.2%)
**ECOG PS**	
0–1	72 (71.3%)
>2	29 (28.7%)
**MMSE score**	
Unknown	9 (8.82%)
<27	37 (36.3%)
>=27	56 (54.9%)
**Neurologic impairment**	
Unknown	2 (1.96%)
No	40 (39.2%)
Yes	60 (58.8%)
**Type of neurologic impairment**	
None/Unknown	41 (40.2%)
Cognitive	5 (4.90%)
Convulsions	2 (1.96%)
Language	9 (8.82%)
More than one	18 (17.6%)
Motor symptoms	20 (19.6%)
Sensorial	4 (3.92%)
Visual	3 (2.94%)
**Type of surgery**	
Unknown	2 (1.96%)
Biopsy	83 (81.4%)
Partial resection	17 (16.7%)
**Number of lesions**	
1	78 (87.6%)
>1	11 (13.3%)
**Tumor volume**	
Median	141.8 cm^2^
Range	16–528 cm^2^

ECOG PS, Eastern Cooperative Oncology Group Performance Status; MMSE, Mini-Mental State Examination.

**Figure 1:**
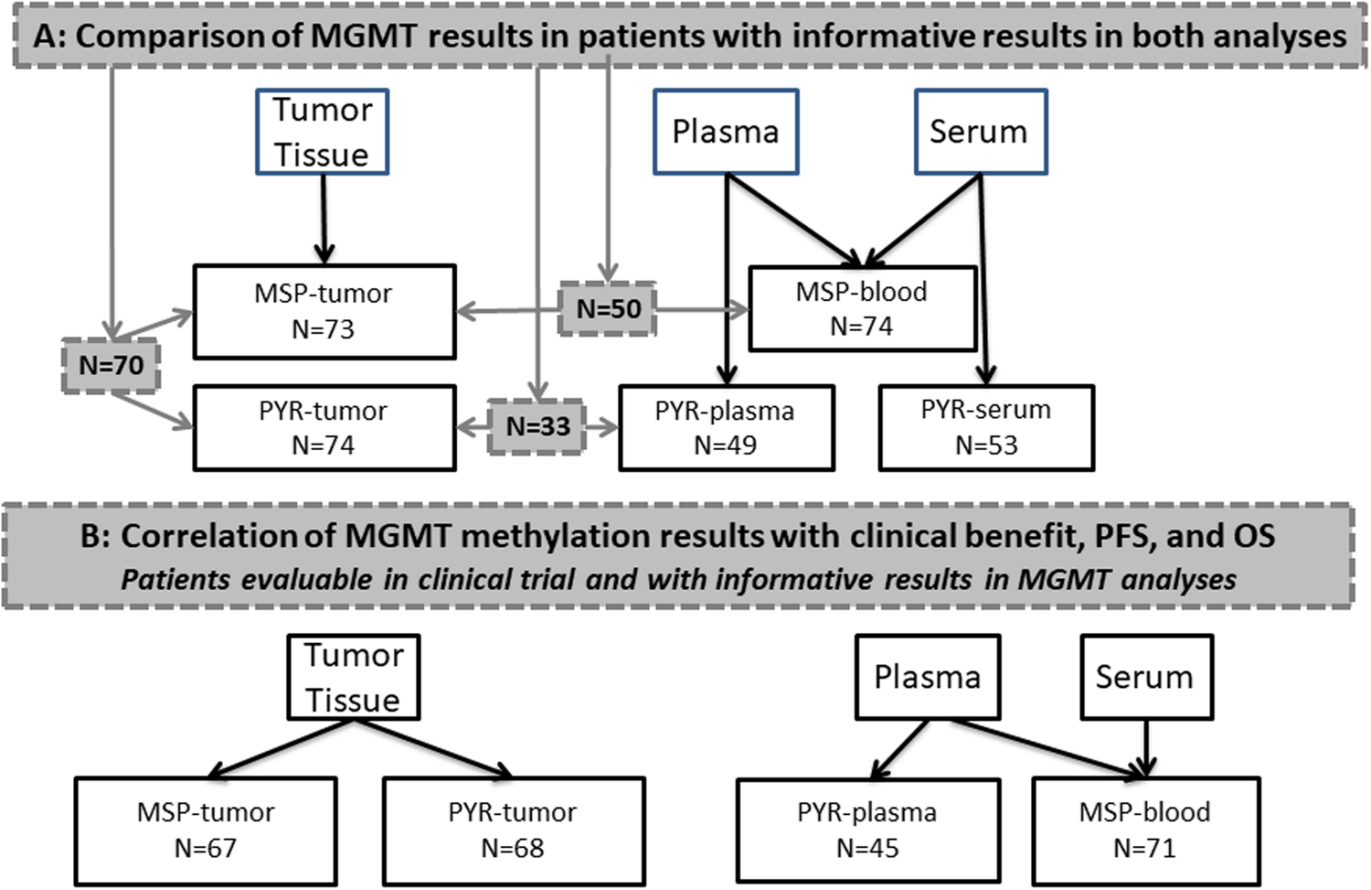
CONSORT diagram showing patients and analyses in the study. (**A)** Numbers in shaded boxes indicate the patients included in the comparisons between the results of different analyses of MGMT methylation. **(B)** Numbers indicate patients evaluable for outcome in the GENOM 009 trial^29^ and with informative results for the MGMT methylation analyses.

We received 83 blood samples and 81 tissue samples from patients included in the GENOM 009 trial for the purpose of assessment of MGMT methylation by MSP. Of the 83 blood samples, six were too hemolyzed and three did not amplify correctly so we obtained results from 74 samples. After the analysis of MGMT methylation in blood (MSP-blood), 64 blood samples remained available for analysis by PYR. Of these 64 samples, 56 were able to be processed for PYR analysis in serum (PYR-serum) and 55 were able to be processed for PYR analysis in plasma (PYR-plasma). Analysis by MSP-blood was informative for 74 patients, while analysis by PYR-serum was informative for 53 patients and by PYR-plasma for 49 patients. Of the 81 tumor samples, eight did not amplify correctly for analysis by MSP in tumor (MSP-tumor). After MSP-tumor, 78 tumor samples were available for analysis by PYR. Four of the eight samples that had not amplified correctly for MSP-tumor were rescued for analysis by PYR in tumor (PYR-tumor). Analysis of MGMT methylation by MSP-tumor was informative for all 73 patients, while analysis by PYR-tumor was informative for all 74 patients. Not all patients had informative results for all the analyses; 70 patients had results for both MSP-tumor and PYR-tumor, 50 had results for both MSP-tumor and MSP-blood, 33 had results for both PYR-tumor and PYR-plasma, and 39 had results for both PYR-tumor and PYR-serum (Fig. 1).

Correlation between MGMT methylation status by each of the analyses and patient outcome in terms of clinical benefit, progression-free survival (PFS), or overall survival (OS) was only calculated for patients who had been randomized in the trial and who had informative results for the analysis of MGMT methylation (Fig. 1).

### Cut-off points for PYR-tumor, PYR-plasma, and PYR-serum

Table 2 depicts percentages of cytosine methylation in tumor tissue, plasma, serum, and normal tissue for each of the five CpG sites analyzed and for the mean of all five, as well as the optimal and minimum cut-off points to identify differences in OS. The optimal cut-off points were 11.4% for PYR-tumor and 3.4% for PYR-plasma. Since the cut-off for PYR-serum (1.6%) was not associated with a significant difference in OS, we ruled out PYR-serum values for further analyses. Minimum cut-offs were 5.0% for PYR-tumor and 3.4% for PYR-plasma. This minimum cut-off was used to classify patients as having MGMT methylation (MET) or not having MGMT methylation (UNMET) by PYR for further analyses.

**Table 2:** Percentage of cytosine methylation in five CpG sites in tumor, plasma, and serum samples by pyrosequencing (PYR).

	CpG 74			CpG 75			CpG 76			CpG 77			CpG 78			Mean of 5 CpG sites		
	PYR-tumor	PYR- plasma	PYR-serum	PYR-tumor	PYR-plasma	PYR-serum	PYR-tumor	PYR-plasma	PYR-serum	PYR-tumor	PYR-plasma	PYR-serum	PYR-tumor	PYR-plasma	PYR-serum	PYR-tumor	PYR- plasma	PYR-serum
**Percentages in PYR-tumor (N** = **68)**, **PYR-plasma (N** = **45)**, **and PYR-serum (N** = **51)**																		
Mean	18.86	7.27	6.19	19.82	8.49	7.23	18.88	9.33	7.19	19.69	8.10	6.57	13.95	8.86	7.60	18.2	8.4	6.9
Median	4.00	2.00	2.00	6.50	3.00	3.00	7.00	3.00	3.00	4.50	2.00	2.00	5.50	3.00	3.00	5.8	2.8	2.6
SD	24.707	18.012	12.972	23.701	18.721	12.702	23.053	19.153	13.166	25.829	19.856	14.459	20.522	20.042	13.688	21.610	19.0	13.3
Optimal cut-off^a^ (p-value^b^)	6 (0.001)	3 (0.02)	1 (0.07)	6 (0.003)	4 (0.18)	2 (0.22)	10 (0.001)	8 (0.002)	2 (0.05)	15 (0.002)	3 (0.01)	1 (0.02)	8 (0.008)	6 (0.06)	1 (0.10)	11.4 (0.002)	3.4 (0.005)	1.6 (0.06)
Minimum cut-off^a^ (p-value^b^)	—	—	—	—	—	—	—	—	—	—	—	—	—	—	—	5 (0.009)	3.4 (0.01)	NC^d^
**Mean of 5 CpG Sites in Normal Tissue**^**c**^																		
	**Colon (N** = **10)**		**Brain (N** = **6)**		**Lymphocytes (N** = **5)**													
Mean	2.4		3.6		2.8													
Median	1.6		4.9		3.0													
SD	2.05		1.02		0.82													

SD, standard deviation; NC, not calculated.

^a^The optimal cut-off was able to identify maximal differences in OS. The minimum cut-off was able to identify any significant differences in OS.

^b^p-value for differences in overall survival.

^c^All values for normal tissue were below the minimum cut-off for PYR-tumor in glioblastoma tissue.

^d^The minimum cut-off for PYR-serum was not calculated because the cut-off was below the median value.

Mean values of the five CpG sites in non-tumor tissue were 2.4% for colon samples, 3.6% for brain samples, and 2.8% for lymphocytes, indicating that some degree of methylation can be found in normal tissue, although all values were under the minimum cut-off identified for glioblastoma tissue (Table 2).

### MGMT methylation by MSP-tumor and PYR-tumor

MSP-tumor identified 35 patients (47.9%) as MET and 38 (52.1%) as UNMET, while PYR-tumor identified 39 (52.7%) as MET and 35 (47.3%) as UNMET. Among the 70 patients with informative results for both MSP-tumor and PYR-tumor, eight (11.4%) identified as UNMET by MSP-tumor were identified as MET by PYR-tumor; conversely, three (4.3%) identified as MET by MSP-tumor were identified as UNMET by PYR-tumor (p < 0.001) (Table 3). Four cases classified as non-evaluable by MSP were evaluable by PYR. MSP-tumor had greater sensitivity (91% vs 78%) and NPV (90% vs 75%) to predict PYR-tumor results than did PYR-tumor to predict MSP-tumor (Table 4).

**Table 3:** Comparison of results of MGMT methylation analysis by MSP-tumor with MSP-blood, PYR-tumor, and PYR-plasma.

Results Compared			Concordance^b^	p^c^
MSP-tumor vs PYR-tumor N = 70^a^				
	MET by MSP-tumor N = 34	UNMET by MSP-tumor N = 36	84.3%	<0.001
MET by PYR-tumor N = 39	31 (44.3%)	8 (11.4%)		
UNMET by PYR-tumor N = 31	3 (4.3%)	28 (40.0%)		
**MSP-tumor vs MSP-blood** **N** = **50**^**a**^			62.0%	0.02
	MET by MSP-tumor N = 26	UNMET by MSP-tumor N = 24		
MET by MSP-blood N = 9	8 (16.0%)	1 (2.0%)		
UNMET by MSP-blood N = 41	18 (36.0%)	23 (46.0%)		
**PYR-tumor vs PYR-plasma** **N** = **33**^**a**^			63.7%	0.23
	MET by PYR-tumor N = 15	UNMET by PYR-tumor N = 18		
MET by PYR-plasma N = 7	5 (15.2%)	2 (6.1%)		
UNMET by PYR-plasma N = 26	10 (30.3%)	16 (48.5%)		

^a^Numbers represent the total number of patients with informative results in both tests being compared.

^b^Percentages indicate the number of cases with identical results in both tests being compared.

^c^χ^2^ or Fisher exact test.

**Table 4:** Sensitivity, specificity, positive predictive value, (PPV), and negative predictive value (NPV) of each test to predict MGMT methylation status as identified by the second test.

Comparison	Parameter	Point Estimates (95% CI)
M-MSP-tumor to predict PYR-tumor N = 70 p < 0.001	Sensitivity	91% (76–98%)
	Specificity	75% (58–88%)
	PPV	78% (62–89%)
	NPV	90% (73–98%)
PYR-tumor to predict MSP-tumor N = 70 p < 0.001	Sensitivity	78% (62–89%)
	Specificity	90% (73–98%)
	PPV	91% (76–98%)
	NPV	75% (58–88%)
MSP-blood to predict MSP-tumor N = 50 p = 0.02	Sensitivity	31% (14–52%)
	Specificity	96% (79–100%)
	PPV	89% (52–100%)
	NPV	56% (40–72%)
PYR-plasma to predict PYR-tumor N = 33 p = 0.23	Sensitivity	38% (15–65%)
	Specificity	76% (50–93%)
	PPV	60% (26–88%)
	NPV	57% (34–77%)

### MGMT methylation by MSP-blood and PYR-plasma compared to MSP-tumor and PYR-tumor

MSP-blood identified 11 patients (14.9%) as MET and 63 (85.1%) as UNMET. PYR-plasma identified 14 (28.6%) as MET and 35 (71.4%) as UNMET. Of 50 patients with informative results in both MSP-tumor and MSP-blood, MSP-blood results were different from MSP-tumor results in 19 (38%) (p = 0.02) (Table 3). Of 33 patients with informative results in both PYR-tumor and PYR-plasma, results differed in 12 (36.3%) (p = 0.23). Sensitivity was low for both MSP-blood (31%) and PYR-plasma (38%), while specificity was higher for MSP-blood than for PYR-plasma (96% vs 76%) (Table 4).

### MGMT methylation and prognosis

**Figure 2:**
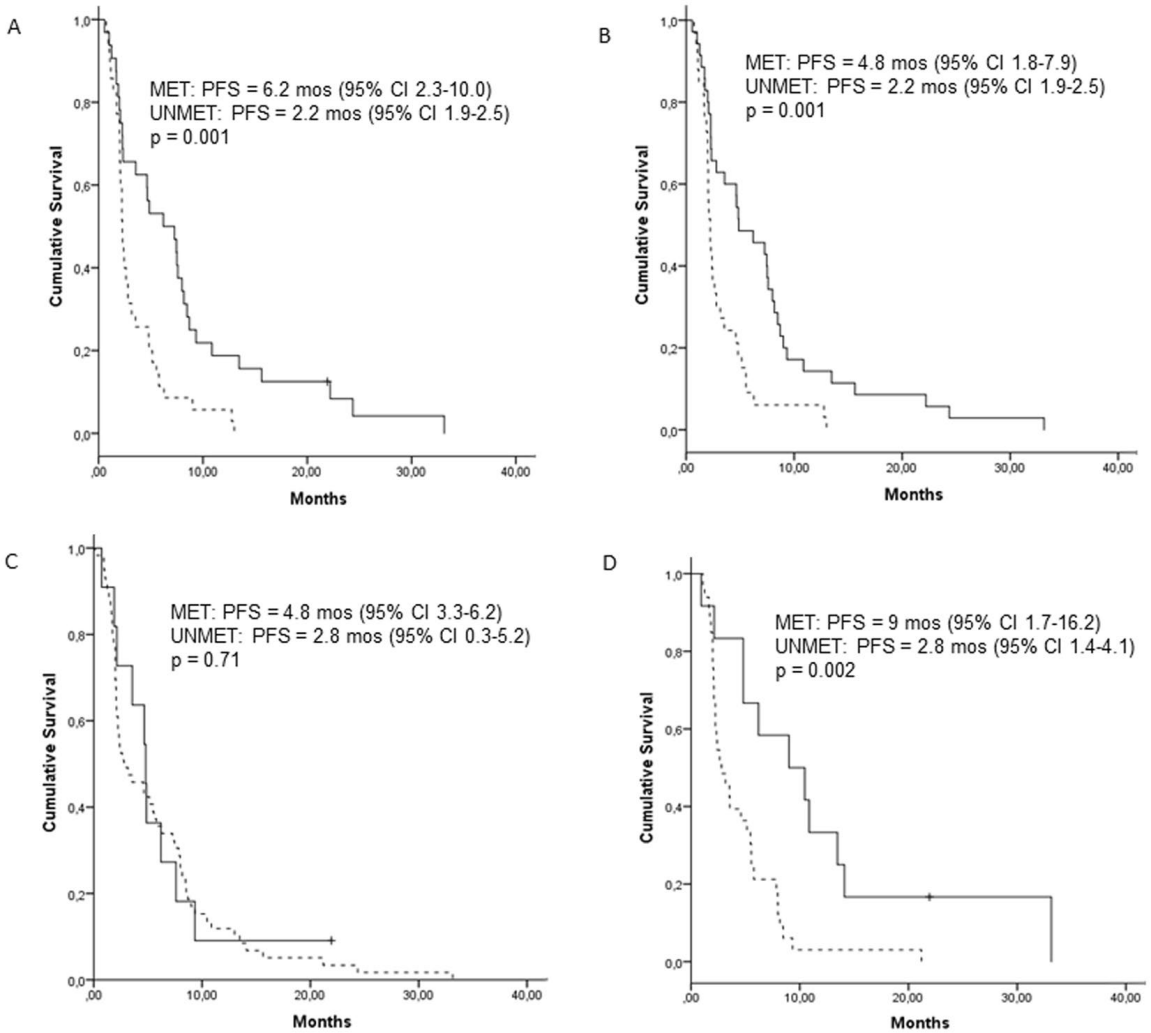
Progression-free survival (PFS) according to the results of the MGMT methylation analyses by (**A)** MSP-tumor, **(B)** PYR-tumor, **(C)** MSP-blood, and **(D)** PYR-plasma. Solid lines indicate methylated MGMT (MET); broken lines indicate unmethylated MGMT (UNMET).

**Figure 3:**
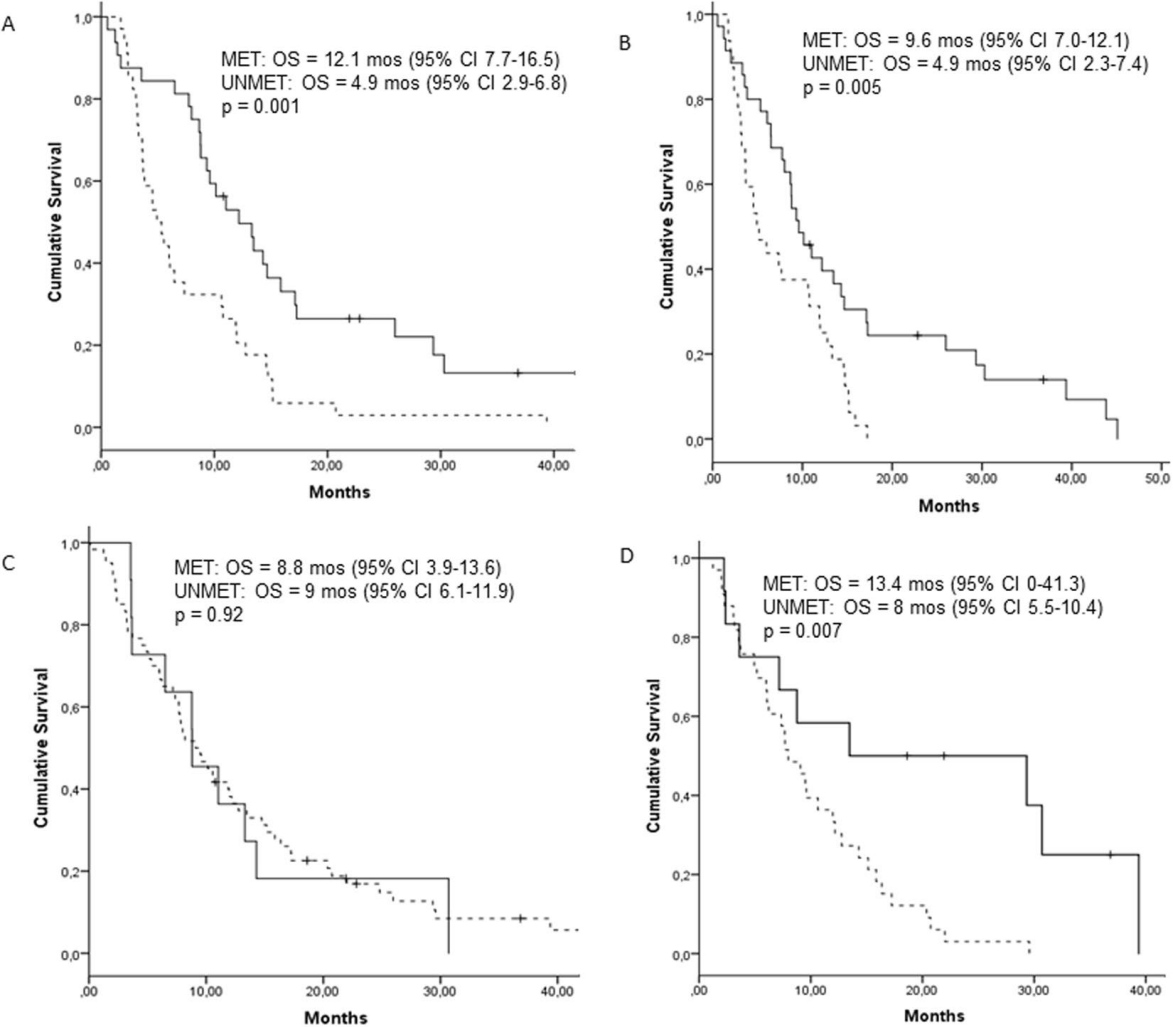
Overall survival (OS) according to the results of the MGMT methylation analysis by (**A**) MSP-tumor, **(B)** PYR-tumor, **(C)** MSP-blood, and **(D)** PYR-plasma. Solid lines indicate methylated MGMT (MET); broken lines indicate unmethylated MGMT (UNMET).

MGMT methylation by MSP-tumor correlated with clinical benefit (p = 0.01), PFS (p = 0.001), and OS (p = 0.001). MGMT methylation by PYR-tumor also correlated with outcomes (p = 0.006, p = 0.001, p = 0.005, respectively). MSP-blood results were not significantly associated with outcome, while PYR-plasma results correlated with PFS (p = 0.002) and OS (p = 0.007) but not with clinical benefit (Table 5 and Figs 2 and 3).

**Table 5:** Clinical benefit, progression-free survival (PFS), and overall survival (OS) according to MGMT methylation status by MSP-tumor, MSP-blood, PYR-tumor, PYR-plasma, and MSP-tumor plus PYR-tumor.

	MSP-tumor		
	MET	UNMET	p
Clinical benefit (N = 63)^a^			0.01
Yes	19 (65.5)	10 (34.5)	
No	11 (32.4)	23 (67.6)	
PFS (N = 67)^a^			0.001
months (95% CI)	6.2 (2.3–10.0)	2.2 (1.9–2.5)	
OS (N = 66)^a^			0.001
months (95% CI)	12.1 (7.7–16.5)	4.9 (2.9–6.8)	
	**MSP-blood**		
	**MET**	**UNMET**	**p**
Clinical benefit (N = 67)^a^			0.17
Yes	7 (21.9)	25 (78.1)	
No	3 (8.6)	32 (91.4)	
PFS (N = 70)^a^			0.71
months (95% CI)	4.8 (3.3–6.2)	2.8 (0.3–5.2)	
OS (N = 71)^a^			0.92
months (95% CI)	8.8 (3.9–13.6)	9.0 (6.1–11.9)	
	**PYR-tumor**		
	**MET**	**UNMET**	**p**
Clinical benefit (N = 65)^a^			0.006
Yes	21 (72.4)	8 (27.6)	
No	8 (27.6)	23 (63.9)	
PFS (N = 68)^a^			0.001
months (95% CI)	4.8 (1.8–7.9)	2.2 (1.9–2.5)	
OS (N = 67)^a^			0.005
months (95% CI)	9.6 (7.0–12.1)	4.9 (2.3–7.4)	
	**PYR-plasma**		
	**MET**	**UNMET**	**p**
Clinical benefit (N = 43)^a^			0.31
Yes	7 (33.3)	14 (66.7)	
No	4 (18.2)	18 (81.8)	
PFS (N = 45)^a^			0.002
months (95% CI)	9 (1.7–16.2)	2.8 (1.4–4.1)	
OS (N = 45)^a^			0.007
months (95% CI)	13.4 (0–41.3)	8.0 (5.5–10.4)	
	**PYR-tumor** + **MSP-tumor**		
	**MET by either test**	**UNMET by both tests**	
PFS (N = 64)^a^			0.01
months (95% CI)	4.6 (2.7–6.6)	2.3 (1.8–2.6)	
OS (N = 63)^a^			0.004
months (95% CI)	9.6 (6.2–12.9)	4.5 (2.5–6.5)	

^a^Clinical benefit, PFS, and OS were analyzed only for patients with informative results in the MGMT methylation analysis. Clinical benefit was analyzed only in patients evaluable for response in the clinical trial. PFS and OS were analyzed for patients included in the trial.

Since four tumor samples did not amplify correctly for analysis by MSP but were able to be rescued for PYR, we speculated that PYR-tumor may yield more informative results than MSP-tumor. However, when we compared OS in the group of patients identified as MET by either method with those identified as UNMET by both methods, results were not superior to those obtained with either method alone (Table 5).

## Discussion

MGMT methylation status is a well-known predictive and prognostic factor in glioblastoma, and its assessment at the time of diagnosis is an important factor both for clinical trials and for deciding on the optimal treatment strategy. In the present study, we have assessed *MGMT* methylation in tumor tissue and in blood by MSP and PYR and compared the reliability of the different analyses. Although results by MSP-tumor and PYR-tumor were not completely identical, concordance was high (84.3%; p < 0.001) and both methods provided reliable results. In contrast, assessment in blood was feasible but less reliable, with a high percentage of false negatives in both PYR-plasma and MSP-blood and a lower level of concordance with the results in tumor tissue.

Although several methods are currently available to assess MGMT methylation, there is as yet no agreement about which test should be considered the “gold standard”^8,30^. Comparative studies often lack a previous study setting the optimal cut-off point for PYR related to clinical benefit^31,32^. To further complicate the comparison of results, each method can interrogate different CpG sites in the MGMT promoter region, and there is still no consensus on how many CpG sites should be explored, which are most highly correlated with prognosis, and whether it is better to select them consecutively or randomly. In the PYR analyses in the present study, we explored CpG sites 74–78, which have a good correlation with the prognosis of patients newly diagnosed with glioblastoma^30–34^ and which overlap almost exactly with those interrogated by MSP^30,35^.

In our clinical practice, we routinely use MSP to analyze MGMT methylation because it is a well-known technique, it is inexpensive when testing only a few samples, it has demonstrated sensitivity, and its results have been associated with outcome in clinical trials. However, MSP is a not an automatized method, making it difficult to standardize, and results may be influenced by tumor heterogeneity and/or a subjective interpretation. Conversely, PYR has been standardized and by giving a quantitative methylation percentage for each analyzed CpG, it is not subject to individual interpretation of results once the cut-off value has been defined. Different cut-off points have been recommended for PYR, with little consensus on the optimal point^32,36,37^, which can vary according to the CpG sites analyzed and which will ultimately depend on its predictive capacity. Reported cut-off values range from 2.7% to 35% and the number of CpG sites analyzed range from four to 62^38^. In the present study, we first defined the cut-off as the one that identified the maximal differences in OS in our patient population. In fact, however, differences in OS started to be seen at lower levels of methylation. Since the clinical objective behind determining MGMT methylation status is to identify those patients most likely to benefit from treatment with TMZ, we then identified the minimum cut-off that identified any differences in OS. This is in line with a recent pooled analysis of 4041 patients from four clinical trials, in whom MGMT methylation was analyzed by quantitative MSP. Lower MGMT methylation conferred some sensitivity to TMZ, leading the authors to recommend that patients in the “gray zone” of MGMT methylation should be considered as methylated in terms of treatment selection^39^.

Using the same criteria, we also determined the cut-off for PYR results in serum and in plasma. Unexpectedly, we found that PYR-serum values were not useful since it was impossible to set a cut-off to identify significant differences in OS. Serum seems to be an inadequate source of tumor derived cfDNA since it is often contaminated during clothing by normal nucleated cells^40^, which also express some degree of MGMT methylation, as we and others^41,42^ have found. In fact, in the present study, we observed a slight degree of MGMT methylation in normal brain, lymphocyte, and colon tissue samples, although all values were under the minimum cut-off identified for glioblastoma tissue.

There was some discordance between MSP-tumor and PYR-tumor results. Eight cases identified as UNMET by MSP were identified as MET by PYR. This could be due to the fact that PYR can detect partial methylation of a given CpG that could be labelled UNMET by MSP^30^. In addition, three cases identified as UNMET by PYR were classified as MET by MSP, perhaps due to an insufficient bisulfite conversion^43^. Moreover, four cases deemed non-evaluable by MSP were evaluable by PYR, probably because less DNA is required to run PYR. These discordances led us to postulate that the two tests could complement each other, so we analyzed differences in OS between patients identified as MET by either test and those identified as UNMET by both tests. However, we found that results were similar to those obtained by each method alone. Therefore, we can conclude that both methods can be reliably used to identify patients as MET or UNMET and we recommend performing PYR after MSP only if MSP does not give an informative result and quality controls fail.

The shedding of cfDNA from tumors to fluids has been known for many years^44^, and multiple studies have shown that tumor-associated alterations can be detected in different fluids at the protein, DNA, and RNA levels using diverse methods, making these liquid biopsies an easier and less invasive way to obtain information on the tumor. Brain tumors have not been an exception to the search for circulating biomarkers^17,26,45^. In the present study, we were able to detect methylation in cfDNA by both MSP-blood and PYR-plasma, but when we examined the capacity of MSP-blood and PYR-plasma to predict MGMT methylation status by the matched tumor assessments, concordance was relatively low, with a sensitivity of 30–40%. The specificity and PPV of PYR-plasma was higher than that of MSP-blood, but false negatives occurred in around 56% of cases by both methods. MGMT methylation in cfDNA has been studied but data on sensitivity, specificity, PPV and NPV are seldom reported^27,46,47^.

The low concordance between results obtained in tissue and plasma could be due to several factors. Firstly, glioblastoma tumors may not shed DNA into the circulation due to the special structure of the blood-brain barrier (BBB) and the blood-brain tumor barrier (BBTB)^48–50^. Even though cfDNA can be detected in the blood of glioblastoma patients^51^, the release of DNA to body fluids has been related to tumor size and aggressiveness^21^. All our patients had enhancing measurable disease on the MRI (Table 1) at the time of blood sample extraction and enrollment in the trial. As contrast enhancement lesions on MRI are due to disruption of the BBB, this suggests that these tumors would logically shed DNA, which would not be prevented by a disrupted BBB. Secondly, even though cfDNA is abundant in plasma, only 0.01–10% comes from tumors, while the rest is released from bone marrow (80–90%), or skin and the gastrointestinal tract (5–10%)^21^. Moreover, cfDNA in fluids is found as small fragments (180–200 bp) that may not contain the regions subjected to analyses of MGMT methylation. On the other hand, cfDNA can also be released to CSF and it seems logical that glioblastoma cfDNA would be more abundant in CSF, simply because CSF is in close contact with tumor cells in CNS tumors^52^ or because cfDNA could be released through areas without the BBB, such as the circumventricular organs and the choroid plexus^53,54^. In fact, diverse mutations detected in CSF could potentially be used for the diagnosis and follow-up of glioma^52,55–59^. Several studies have compared results in serum and CSF and found that sensitivity in CSF seems to be higher than in serum^47,55^. However, increased intracranial pressure can be a limitation for the routine assessment of CSF in patients with intracranial hypertension because lumbar puncture carries the risk of cerebral herniation in these cases^60^. Finally, the detection of cfDNA is also limited by the sensitivity of the technique, as semi-quantitative PCR detects only around 1% of cfDNA. Nevertheless, if more sensitive methods for detecting cfDNA in fluids are developed, these limitations could be minimized in the near future^61^.

In summary, both PYR and MSP are reliable methods for detecting MGMT methylation in tumor tissue and can be useful for identifying patients likely to benefit from TMZ. MSP can be recommended for use with small numbers of patients, while PYR is more efficient in large numbers of cases and, moreover, can serve as a useful back-up when MSP results are inconclusive^32,62^. In contrast, both methods are imperfect for analysis in blood.

The main limitation of our study is the low number of paired tissue-blood samples that could be compared. Nevertheless, we are convinced that the identification of reliable circulating biomarkers can lead to major changes in our approach to brain tumors, making it essential to continue to search for circulating biomarkers with high sensitivity, specificity, PPV, and NPV. We therefore recommend that biomarker analysis be incorporated into large clinical trials.

## Methods

### Patients and samples

Patients proceeded from the GENOM 009 trial^29^, in which unresected glioblastoma patients were randomized to add or not bevacizumab to two cycles of TMZ before radiation therapy with concomitant and adjuvant TMZ. Overall response rate, PFS and OS were primary endpoints of the trial. Results showed a non-significant trend towards improvement in PFS and OS for those patients treated with bevacizumab. A secondary endpoint was the study of MGMT methylation status in blood as compared with tissue in these patients. Before patient inclusion, formalin-fixed paraffin-embedded (FFPE) tumor tissue blocks and paired blood samples were obtained from patients and sent to our center for assessing the methylation status of *MGMT*.

In addition, we obtained 21 samples of non-tumor tissue proceeding from normal brain (6), lymphocytes (5), and colon (10). MGMT methylation status in these samples was analyzed by PYR.

Written informed consent was obtained from all patients before registration, both for participation in the trial and for molecular tests. The study was approved by the Ethics Committee of the Hospital Germans Trias i Pujol, Badalona, Spain and by the Ethics Committees of each of the participating hospitals. All research was performed in accordance with relevant guidelines/regulations.

### DNA extraction from tissue and serum/plasma samples

Genomic DNA was extracted from FFPE tissue after macrodissection was performed to assure more than 80% of tumor cells. The material was deparaffinated and DNA obtained using the QIAamp® DNA micro kit (Qiagen, Hilden, Germany). Venous blood (10 mL) was drawn from each patient into Vacutainer tubes containing SST (serum-separating tube) gel and clot activator (Becton Dickinson, NJ, USA), and for plasma in Vacutainer tubes containing k2E (EDTA). Serum and plasma were isolated after centrifugation at 2,500 rpm for 10 min at room temperature and were stored at −20 °C until analysis. The QIAmp® Blood Mini-Kit (Qiagen) was used to obtain DNA according to the manufacturer’s instructions.

### DNA bisulfite conversion

DNA methylation in CpG island of *MGMT* (Genbank accession number NG_052673.1) was determined using two different methods, MSP and PYR. The first step in both methods was DNA bisulfite conversion. The bisulfite treatment converts unmethylated – but not methylated – cytosines, to uracil. Briefly, a total of 250 ng of genomic DNA was modified using EZ DNA Methylation-Gold ™ kit (Zymo Research, Ecogen, Madrid), following the manufacturer’s instructions, and recovered in a final volume of 20 μl.

### MGMT methylation assessment by MSP

Bisulfite-converted DNA (2 μl) was amplified using specific primers (previously described for CpG sites 74–78^63^) for methylated and unmethylated DNA independently, using HotStart®Plus DNA polymerase (Qiagen, Izasa, Spain) and following the manufacturer’s instructions. Cycling conditions were 5 min 95 °C, followed by 42 cycles of (30′′ 95 °C, 30′′ 59 °C, 30′′ 72 °C) and 5 min at 72 °C. PCR reactions (15 μl) were analyzed on a 2% agarose gel stained with ethidium bromide or Syber Safe. Commercial methylated DNA and unmethylated DNA (Zymo Research, Ecogen, Spain) served as positive controls for methylated and unmethylated PCR reactions.

A sample was considered methylated when a band was observed in PCR (in duplicate) with methylated primers. When no band was observed in either tube the sample was assessed as “not evaluable”. When both duplicates gave different results, the test was repeated for that sample in triplicate.

### MGMT methylation assessment by PYR

Bisulfite-converted DNA (2 μl) was used to amplify the MGMT promoter with the primers provided by the Pyromark®Q24 CpG MGMT kit. A PCR was set up with the reactives and conditions given by the PyroMark®PCR kit. The same CpG sites (74 to 78) analyzed by MSP were analyzed by PYR. Briefly, PCR conditions for tissue DNA were 5 min 95 °C, followed by 45 cycles of (20′′ 95 °C, 30′′ 53 °C, 20′′ 72 °C) and 5 min at 72 °C. In plasma and serum, the number of cycles was increased to 50. PCR products (10 μl) were attached to Streptavidina Sepharose perls (GE Healthcare, Buckinghamshire, UK) and the template strands were purified in the Pyromark Q24 Vaccum Workstation. The purified templates were incubated for 20 min with the sequencing primer provided by the kit and run in the instrument Qiagen Pyromark Q24 System with the reactives of Pyromark Gold Q24 Reagents. All the steps were followed according to the Pyromark Q24 user manual. Sequencing conditions and analysis of results were performed with the Pyromark Q24 sofware 2.0.8. Commercial unmethylated DNA was used to calculate baseline (n = 13).

In case of discordant results between PYR and MSP, MSP was repeated to confirm the discordance.

### Determination of cut-off points for PYR-tumor, PYR-plasma, and PYR-serum results

PYR results were delivered as a percentage of cytosine methylation for each CpG site assessed in tissue, plasma, and serum. We created a new variable using the overall mean of all five CpG sites (Table 2). We calculated the cut-off that yielded maximal differences in OS for each CpG and for the mean (optimal cut-off point) for PYR-tumor, PYR-plasma, and PYR-serum. However, despite having identified the optimal cut-off point for maximal differences in OS, we reasoned that many patients with MGMT methylation status below that cut-off could still benefit from treatment because of a certain degree of methylation. We therefore identified the minimum cut-off point for the mean of all five CpG sites that yielded a significant difference in OS. This cut-off was used for further analyses. If the value was higher than the cut-off, a sample was classified as MET; if it was equal to or lower than the cut-off, a sample was classified as UNMET. All cut-off points were calculated with the Maximally Selected Rank Statistics (Maxstat package of R, version 1.1.442) and confirmed with the Kaplan-Meier method and log-rank test.

### Statistical analyses

The epiR package of R was used to calculate the sensitivity, specificity, PPV, and NPV of the different analyses to predict MGMT methylation status as assessed by other analyses. Since in this case, none of the analyses could be considered the established gold standard against which to compare the others, we performed bilateral comparisons of PYR-tumor vs MSP-tumor, MSP-tumor vs PYR-tumor, PYR-plasma vs PYR-tumor, and MSP-blood vs MSP-tumor.

Categorical variables were compared with the χ^2^ test or the Fisher’s exact test. All patients who started treatment were included in the analyses. Response to neoadjuvant therapy was evaluated by RANO criteria, after two cycles of therapy and before radiation therapy. For the purpose of analysis, responses were grouped as clinical benefit (stable disease, partial response, or complete response) and no clinical benefit (progression)^64^. PFS was defined as the time from inclusion to the first documented progression or death from any cause, while OS was defined as the time from inclusion to death from any cause. Patients who were still progression-free or alive at the date of last contact were censored. Median PFS and OS were calculated with the Kaplan-Meier method and compared using the log-rank test. All statistical tests were two-sided and significance was set at 0.05. These analyses were performed with SPSS v24.0 (IBM).

## Data Availability

The datasets generated during and/or analyzed during the current study are available from the corresponding author on reasonable request.
